# Canonical and retinal size in visual working memory

**DOI:** 10.3758/s13423-026-02913-8

**Published:** 2026-04-21

**Authors:** William L. Gronewald, Vanessa G. Lee, Roger W. Remington

**Affiliations:** Department of Psychology, University of Minnesota, S504 Elliott Hall, Minneapolis, MN 55455 USA

**Keywords:** Visual working memory, Canonical size, Attention, Change detection

## Abstract

Visual working memory (VWM) is key to many daily tasks, such as remembering visual information about traffic when crossing a busy street. Despite extensive research, the extent to which VWM abstracts out sensory properties not relevant to identification, such as object size, remains unclear. In three experiments, we examined how object size affects VWM, with size defined in two ways: retinal size, referring to the image’s size on the screen (small or large photos), and canonical size, referring to the typical size of objects in the real world, from big (e.g., a tower) to small (e.g., an egg). Experiments 1 and 2 tested memory for real-world objects, classified into four types based on their photo size and canonical size. VWM was better for large rather than small photos—a retinal-size effect—and for canonically small than big objects—a canonical-size effect. These effects were stronger when participants remembered a mix of different-sized objects than when all objects in a display were of the same size. Experiment 3 tested memory for colored squares that were either big or small on the screen. Size had no effect when displays consisted of colored squares of the same size, but big squares were remembered better when mixed with small squares. These results suggest that seemingly irrelevant sensory properties affect VWM, favoring objects that stimulate more neurons. The effect is stronger when size conditions are mixed, indicating that retinally larger objects are better attended.

## Introduction

Many daily activities require us to hold visual images in memory for durations ranging from milliseconds to days or years. Previous research divides visual memory into three categories: sensory (or iconic) memory, visual working memory (VWM), and visual long-term memory (VLTM; Phillips & Christie, 1977). Iconic memory, lasting a few hundred milliseconds, is tied to eye position (Coltheart, 1980), and is easily disrupted by new visual input (Becker et al., 2000). Conversely, VLTM stores information for extended periods, allowing us to remember familiar faces or places visited. VLTM exhibits massive capacity (Brady et al., 2008) and contains conceptual information as well as visual details such as color and orientation. Interestingly, VLTM retains “canonical” object size. Naturally large objects (e.g., houses) are imagined as occupying more space, whereas naturally small objects (e.g., eggs) occupy less (Konkle & Oliva, 2011). Canonical size is unlikely a purely visual property, as evidenced by its representation in adults with congenital blindness (Szubielska et al., 2025). Its amodal nature raises questions about how it affects performance in short-term tasks.

VWM, an intermediate memory between iconic memory and VLTM, retains information for a few seconds and can store up to four objects with their associated features (Luck & Vogel, 1997). VWM appears to abstract away certain low-level features. Unlike iconic memory, VWM is resistant to changes in eye position: test displays can be recognized even under changes in retinal locations, as long as the spatial configuration remains consistent (Irwin & Andrews, 1996; Jiang et al., 2000). However, VWM retains features of sensory memory: It can be disrupted by irrelevant visual input or the test display (Lamme, 2003; Makovski & Jiang, 2008). In addition, people remember the displayed size of objects, as changes in photo size between encoding and retrieval impair recognition (Jolicoeur, 1987). VWM is also sensitive to the similarity between encoded stimuli: Items from mixed categories such as faces and scenes are better remembered than those from a single category (Cohen et al., 2014), though highly similar features (e.g., colors) are remembered with greater precision than dissimilar ones (Lin & Luck, 2012).

Brain imaging studies have shown that early visual areas and high-level category areas are both activated during VWM retention, consistent with the behavioral evidence that VWM retains both sensory and abstract components (Todd & Marois, 2005; Tong, 2003). However, it is unclear whether task-irrelevant visual properties affect how well stimuli are remembered. One such property is retinal size, operationalized as the surface area an object occupies when viewed from a fixed distance. VWM is known to retain sensory properties intrinsic to objects in an image, such as color. Retinal size is a different kind of sensory property, an extrinsic factor. It reflects how the viewer experiences an image in context and is irrelevant to intrinsic properties necessary for identification, raising the possibility that VWM is invariant to object size. Alternatively, larger photos stimulate more neural activity in perceptual areas, which could improve memory. In change-detection paradigms, larger photos could also produce a larger “change” signal in sensory areas that enhance the comparison process.

Retinal size was investigated in VLTM by Masarwa et al. (2022), who asked participants to view more than 100 photographs subtending from 3º to 21º. In a later surprise memory task, participants identified old photographs among new ones. Although size was task-irrelevant, larger photos were recognized better than smaller ones. Masarwa et al. attributed this to greater neural resources for larger images. However, Masarwa et al. tested only VLTM, not VWM, and they did not test the effect of canonical size on memory.

The goal of this study was to examine how retinal size influences VWM for objects that are canonically big or small. Experiments 1 and 2 asked participants to remember objects that were either canonically big (e.g., a piano) or small (e.g., an egg), each occupying either a small or large area on the screen to vary retinal size. Experiment 3 used large or small colored squares, eliminating the need to remember object details and isolating the sensory component by removing canonical size.

We tested several hypotheses. First, as in VLTM, VWM may be sensitive to congruency between retinal and canonical size, such that canonically smaller objects are better remembered when occupying a smaller area, whereas canonically bigger objects are better remembered when occupying a larger area (size-congruency hypothesis). Alternatively, because they project to more neurons and produce a larger change signal, retinally large objects may be better remembered than small objects regardless of canonical size (sensory VWM hypothesis). This hypothesis contrasts with the *abstract* VWM hypothesis, which postulates that VWM is unaffected by retinal size. We also examined whether objects with different canonical sizes differ in memorability (Bainbridge et al., 2013), favoring either canonically big objects due to their imposing nature or small objects due to their manipulability in everyday life (canonical-size hypothesis).

## Experiment 1

**Fig. 1:**
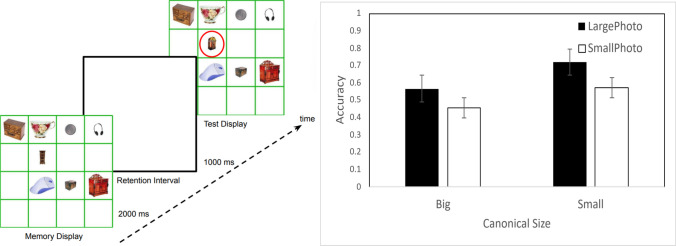
Stimuli and results from Experiment 1. Left: Sample trial sequence. The red circle indicating the changed object is shown here for illustrative purposes only. Right: Change-detection accuracy. Error bars show ± 1 *SEM* across participants. Images are from Talia Konkle’s image database (https://konklab.fas.harvard.edu/#). (Color figure online)

Experiment 1 tested how retinal and canonical size interact to affect VWM for common objects. Participants completed a change-detection task between two displays separated by a 1-s retention interval (Fig. 1, left). The memory array contained eight objects, two in each of four possible size conditions—canonically small or large, and occupying either a small area (3.14º × 3.14º) or a large area (6.29º × 6.29º). The test array was identical, except for one object, which belonged to the same size condition as the one it replaced. The task was to identify the changed object by clicking on it. We examined how memory performance varied across the four size combinations.

### Method

#### Sample size

There were no previous studies on retinal and canonical size effects in VWM. Therefore, we predetermined a sample size of 16 participants for each experiment based on previous studies of VWM for objects (Cohen et al., 2014; Jiang et al., 2016), which used sample sizes of 10 to 20 and reported effect size Cohen’s *d* from 0.96 to 1.22. At *N* = 16, G*Power (Faul, 2007) showed that the experiment could detect an effect of *d* = 0.75 with a power of.80 in a two-tailed paired-samples *t* test with an alpha of.05. As reported below, the observed effect sizes exceeded this estimate. The three experiments provided conceptual replications, reducing the likelihood that the significant effects were spurious.

#### Participants

The 16 participants in Experiment 1 included one man and 15 women, with a mean age of 19.2 years (*SD* = 0.94). One additional participant completed the study after we reached the targeted enrollment; their data were not included in the analysis. All participants met the inclusion criteria: age 18–45 years, normal visual acuity (self-report), passed an Ishihara color-blindness test (administered in a booklet), naïve to the purpose of the study, and no history of neurological or psychiatric conditions. Participants received cash or extra credit for their time. The study was approved by the University of Minnesota Institutional Review Board.

#### Equipment

Participants were tested individually in a quiet room with normal, well-lit interior lighting. They sat at an unrestrained distance of approximately 40 cm from a CRT monitor (19-in.; spatial resolution 1,024 × 768 pixels; vertical refresh rate: 75 Hz). The experiment was programmed with MATLAB (www.mathworks.com) and Psychtoolbox (Kleiner et al., 2007).

#### Stimuli

Visual stimuli were drawn from a set of 400 objects from Dr. Konkle’s image database (https://konklab.fas.harvard.edu/#). Konkle and Oliva (2011) categorized the canonical size of 200 objects as big (e.g., a piano) and the other 200 as small (e.g., an egg). The grid in which the objects were presented was either 3.14º × 3.14º (small) or 6.29º × 6.29º (large). Stimuli appeared in a 4 × 4 green-line grid, with each cell measuring 7.19º × 7.19º.

#### Procedure and design

On each trial, participants saw eight objects in randomly chosen locations inside the grid for encoding. To allow adequate encoding of these complex stimuli, we presented the objects for 2 s. Because participants likely made saccades during encoding, our findings reflect VWM for items encoded during active viewing rather than purely presaccadic storage. There were two objects from each size condition: canonically big with a large photo size, canonically big with a small photo size, and canonically small with a large or small photo size. The memory load of eight exceeded VWM capacity, a design choice intended to increase attentional competition among objects of different sizes (Luck & Vogel, 2013).

After a 1-s blank retention interval, the objects reappeared, with one object changed (the probe object) from the same size condition as the object it replaced (Fig. 1, left). Participants clicked the object that differed from the first display, requiring change localization based on object memory. Clicks within 6.74º of the object’s center were scored as correct, and accuracy was the only measure; speed was not a factor. To maintain task engagement, each correct response was followed by three rising beeps, whereas an incorrect response was followed by a low buzz. After four practice trials, participants completed 12 blocks of 32 trials each, with the opportunity to take a break before each block. Practice objects were not used in the main experiment.

The selection of stimuli was counterbalanced between participants. The big and small object sets were each randomly numbered from 1 to 200. Even-numbered participants (e.g., the second, fourth) saw odd-numbered objects that were canonically small and even-numbered objects that were canonically big, whereas odd-numbered subjects saw the opposite. Each of the 384 trials used a distinct probe object, and the seven unchanged objects were unique and randomly chosen from the remaining objects on that trial. As in other VWM studies, we did not measure visual imagery or task strategies. However, the brief retention interval made it likely that performance relied primarily on working memory representations rather than later stages of processing, such as imagery or VLTM.

## Results and discussion

Retinal and canonical size conditions were intermixed on the display in Experiment 1. Under this condition, both canonical and photo size affected memory performance (Fig. 1, right). An analysis of variance (ANOVA) using retinal size and canonical size as within-subject factors showed significant main effects of retinal size*, F*(1,15) = 35.635, *p* <.001, ${\eta }_{p}^{2}$ =.704, with better memory for large photos, and canonical size, *F*(1,15) = 353.355, *p* <.001, ${\eta }_{p}^{2}$ =.959, with better memory for canonically smaller objects. These factors did not interact, *F*(1,15) = 1.927, *p* =.185, ${\eta }_{p}^{2}$ =.114, indicating an advantage for retinally large photos regardless of whether objects were canonically big or small.

We did not find evidence for a congruency effect between canonical and photo size. The size-congruency account predicts that for canonically small objects, memory should be better when they are presented at retinally smaller sizes. We found the opposite in our data: Canonically small objects were better remembered when presented at retinally larger sizes. In fact, the advantage for retinally larger stimuli was numerically greater for canonically small objects. This pattern of results strongly argues against the size-congruency account.

While the photo-size effect suggests that irrelevant sensory features affect VWM, it is also consistent with an attentional competition account. Larger photos, being more imposing, were preferentially attended, resulting in better memory than smaller photos. Likewise, canonically smaller objects may enjoy an attentional advantage when mixed with canonically larger objects. Experiment 2 tested whether these effects still held when objects of different sizes were not mixed on a display.

## Experiment 2

**Fig. 2:**
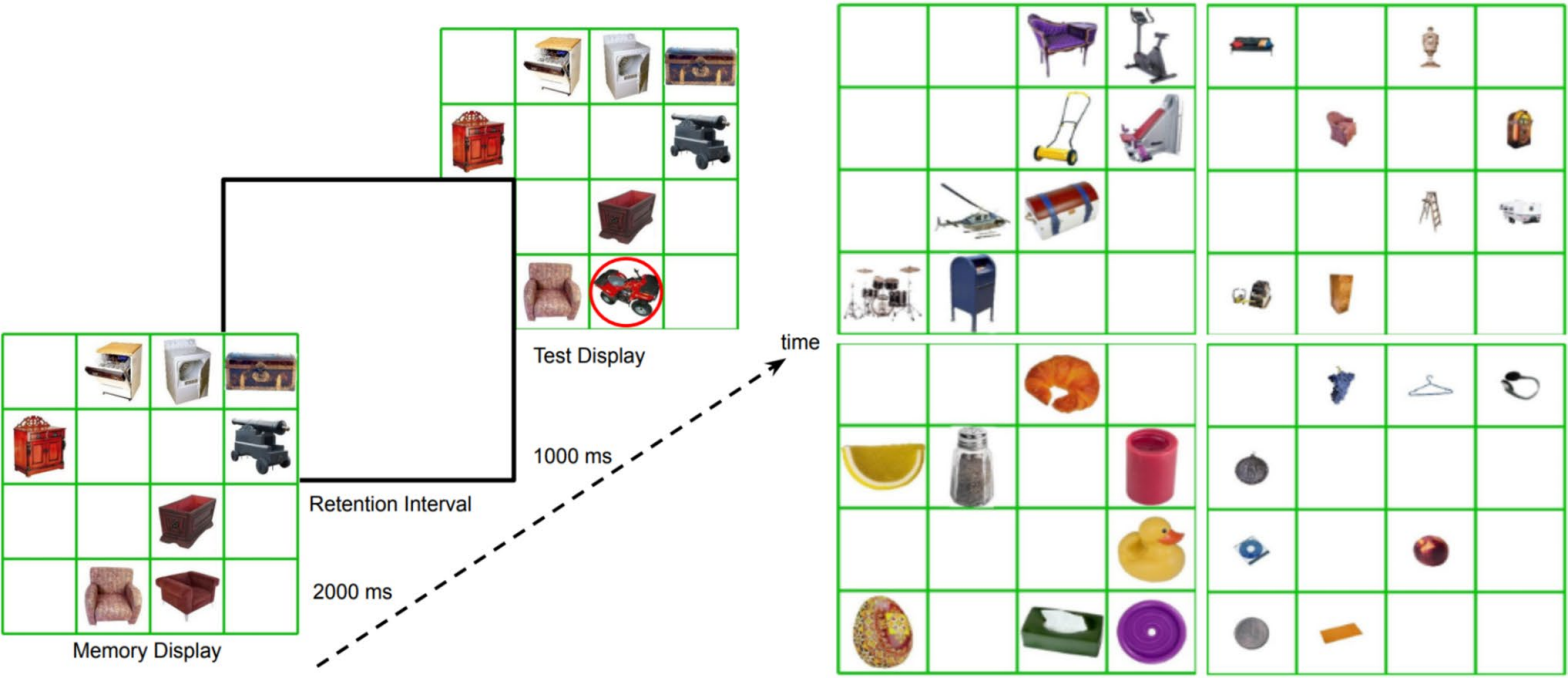
Procedure and stimuli of Experiment 2. Left panel: Sample trial sequence. Note that all objects on the display belonged to a single size condition (e.g., canonically large objects in a large photo). The red circle indicating the changed object is shown here for illustrative purposes only. Right panel: Four displays showing the four possible size conditions. Images are from Talia Konkle’s image database (https://konklab.fas.harvard.edu/#). (Color figure online)

This experiment aimed to examine the effects of retinal and canonical size on VWM while minimizing attentional competition among objects of different sizes. The eight objects in a single display all belonged to the same retinal and canonical size condition, minimizing direct competition between large and small objects. The four size conditions were tested on different trials (Fig. 2). If the retinal and canonical size effects observed in Experiment 1 were driven mainly by attentional competition, then these effects should diminish in Experiment 2.

### Method

#### Participants

Sixteen new participants from the same participant pool were tested in Experiment 2. There was one man and 15 women with a mean age of 19.2 years (*SD* = 0.96). One additional participant was excluded due to low accuracy below 3 standard deviations of the group mean (3 *SD* cutoff was 27%; the excluded data had 22%). A second additional participant completed the study after we reached the targeted enrollment; their data were not included.

#### Stimuli, procedure, and design

This experiment was identical to Experiment 1, except that instead of mixing the four size conditions within a display, each trial displayed eight objects from the same size condition. The 32 trials of each block were randomly and evenly divided across the four size conditions. Figure 2 shows the trial sequence (left panel) and sample displays (right panel).

### Results

**Fig. 3:**
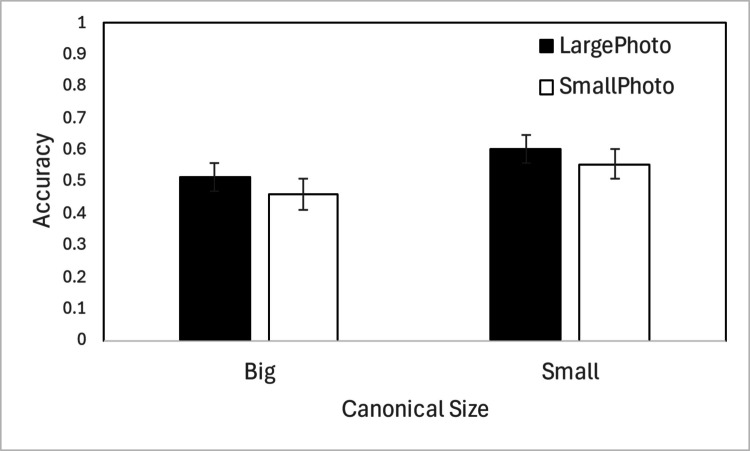
Results from Experiment 2. A single trial contained eight objects from the same size condition. Error bars show ± 1 *SEM* across participants

Figure 3 displays results from Experiment 2, in which retinal and canonical size were homogeneous within displays. An ANOVA with retinal size and canonical size as within-subject factors showed significant main effects of retinal size *F*(1,15) = 11.665, *p* =.004,${\eta }_{p}^{2}$ =.437, indicating better memory for large photos, and canonical size, *F*(1,15) = 75.277, *p* <.001, ${\eta }_{p}^{2}$ =.834, indicating better memory for canonically small objects. These two factors did not interact, *F*(1,15) =.114, *p* =.74, ${\eta }_{p}^{2}$ =.008, meaning for both canonically large and small objects, memory was better if the object appeared in a larger photo.

To examine how size homogeneity affected VWM, we conducted a mixed-factors ANOVA with experiment (mixed display as in Experiment 1 vs. homogeneous display as in Experiment 2) as a between-subjects factor and retinal size and canonical size as within-subjects factors. We found a significant main effect of retinal size, *F*(1,30) = 46.882, *p* <.001, ${\eta }_{p}^{2}$ =.610, showing better memory for large photos, and a significant main effect of canonical size, *F*(1,30) = 314.640, *p* <.001, ${\eta }_{p}^{2}$ =.913, showing better memory for canonically small objects. There was no main effect of experiment, *F*(1,30) = 2.462, *p* =.127, ${\eta }_{p}^{2}$ =.076, suggesting that mixing stimuli within a display did not significantly enhance overall memory. However, experiment interacted with retinal size, *F*(1, 30) = 8.480, *p* =.007, ${\eta }_{p}^{2}$ =.220, and with canonical size, *F*(1,30) = 11.443, *p* =.002, ${\eta }_{p}^{2}$ =.276: the advantages for large photos and canonically small objects were greater in Experiment 1 (mixed displays) than in Experiment 2 (homogeneous displays). The three-way interaction was not significant, *F*(1,30) = 1.613, *p* =.214, ${\eta }_{p}^{2}$ =.051.

### Discussion

Although Experiment 2 no longer mixed objects of different sizes within a display, we observed qualitatively similar results as Experiment 1: better memory for large photos and canonically small objects. The main effects were weaker, but nonetheless large photos and canonically small objects were still remembered significantly better even when attentional competition was minimized. These factors did not interact, again providing no evidence for a congruency effect.

That the retinal-size effect persists with homogeneous displays suggests irrelevant sensory properties are routinely represented in VWM and not solely due to attentional competition. While the advantage for large photos could reflect the recruitment of more visual neurons and a larger change signal, the advantage for canonically small objects is less clear. Small objects are more consistent with their real-life size than even the large photos of canonically large objects, so some form of canonical congruency cannot be ruled out. Alternatively, the small objects may have been more dissimilar from each other, or, because we often manipulate small objects, we may have better internal representations of them than of big objects. Beyond the empirical observation, our data do not speak to the underlying cause of the memory advantage for canonically small objects.

## Experiment 3

In addition to stimulating more neural activity, larger photos alter spatial frequency, potentially allowing finer detail to be processed. Experiment 3 tested the role of visual detail by using large and small colored squares, presented either in intermixed or single-size displays. If the retinal-size effect reflects general factors, such as a larger area containing the “change” signal, then it should generalize to colored squares. Alternatively, the lack of visual details may allow colors to be remembered equally well regardless of size. Similarly, if larger stimuli have an intrinsic attentional advantage, then the retinal-size effect may be stronger when large and small colors were intermixed rather than separated.

### Method

#### Participants

Sixteen new participants from the same pool as the earlier experiments completed Experiment 3. There were five men and 11 females with a mean age of 21.0 years (*SD* = 2.68). In addition to meeting the inclusion criteria of the first two experiments, all participants successfully passed a color blindness test.

#### Stimuli

Visual stimuli included differently colored large (6.29º × 6.29º) or small (3.14º × 3.14º) squares. There were nine possible colors of squares including red, blue, yellow, green, cyan, orange, black, purple, and pink. Squares were presented in a 4 × 4 grid, with each grid cell having an area of 7.19º × 7.19º.

#### Procedure and design

Following four practice trials, participants completed ten blocks of 32 trials each, with the opportunity to take breaks between blocks. On each trial, participants saw six different colored squares in randomly chosen locations inside the 4 × 4 grid for encoding (2 s). After a 1-s blank retention interval, the test display appeared with one square having changed color. Squares could not change into a color already present on the encoding display. Participants clicked on the one that they thought had changed, after which they received the same auditory feedback as in the other experiments.

This experiment tested both heterogeneous and homogeneous-size displays. In half of the trials, all the squares were the same size, either all small or all large. In the other half of trials, size conditions were intermixed: Three squares were large and three were small, with either one of the large squares or small squares changing color. The trial size conditions were randomized in each block.

### Results and discussion

**Fig. 4:**
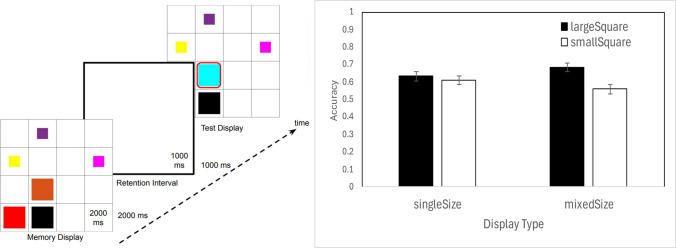
Stimuli and results from Experiment 3’s color VWM. Left: A sample trial sequence. The red frame outlining the changed color is shown here for illustrative purposes only. Right: Color memory accuracy. Error bars show ± 1 *SEM* across participants. (Color figure online)

Large squares were better remembered than small squares, but only when they were intermixed within a display (Fig. 4, right). An ANOVA using encoding display (homogeneous vs. heterogeneous sizes) and size tested (large or small) as within-subjects factors showed no main effect of display homogeneity, *F*(1,15) = 0.004, *p* =.951, ${\eta }_{p}^{2}$ =.000, suggesting that overall performance was independent of whether large and small colors were intermixed. That is, size heterogeneity did not enhance color memory. There was a significant main effect of square size, *F*(1,15) = 33.488, *p* ≤.001, ${\eta }_{p}^{2}$ =.691, with large squares remembered better than smaller ones. However, this effect was qualified by a significant interaction between display type and square size, *F*(1,15) = 17.531, *p* ≤.001, ${\eta }_{p}^{2}$ =.539: square size mattered more in mixed-size displays than in single-size displays.

Follow-up *t* tests showed that when large and small squares were intermixed, large squares were remembered better than small squares, *t*(15) = 7.01, *p* <.001, *d* = 1.75. However, when each display contained only large or only small squares, performance did not differ by size, suggesting that larger squares were not intrinsically easier to remember, *t*(15) = 1.38, *p* = 0.188, *d* = 0.35.

Thus, while the retinal-size effect generalized to VWM for colors, it was primarily driven by attentional competition between large and small squares. When size conditions were separated into different trials, large squares were not remembered significantly better. This suggests that the overall area containing the “change” signal was not the main driver of the retinal-size effect observed with objects.

## General discussion

Three experiments examined the effects of canonical and retinal size on visual working memory (VWM) for objects (Experiments 1 and 2) and colors (Experiment 3). For both canonically big and small objects, large photos led to better object memory than small photos, consistent with a previous study of incidental VLTM (Masarwa et al., 2022). Our study extended Masarwa et al. (2022) by testing shorter retention intervals, manipulating canonical size and display mixing, and including a color control, yielding additional insights into how size affects memory. Because participants likely made saccades during the 2-s-long encoding, our findings reflect VWM for items encoded during active viewing rather than purely presaccadic storage.

We found that the effect of retinal size was significant even when photo sizes were uniform but was stronger when large and small photos were intermixed within a display. A similar effect was observed for color VWM, with better memory for large colored squares than for small ones, but only when the colors were intermixed. These findings suggest that retinal size (i.e., photo size) influences VWM in two ways: through attentional competition favoring larger items and through sensory modulation that enhances memory for retinally larger objects. The attentional effect applies to both objects and colors, whereas sensory modulation primarily benefits memory for objects.

The hypothesized role of attention is consistent with the idea that salient stimuli—such as larger images—attract attention more effectively than small images, though the generality of this effect remains uncertain. We extend prior findings on sensory representation in VWM, such as Jolicoeur (1987), who showed that mismatches in size between encoding and testing disrupted memory. In our study, the test item matched the memory object in photo size, yet VWM was worse for objects in smaller photos. This suggests that size is an intrinsic property of representation in VWM—not only do we remember photo size (Jolicoeur, 1987), but size also modulates how well objects are remembered, even when task-irrelevant.

Although heterogeneity in retinal size biases attentional competition, it did not improve overall memory in our study. This contrasts with Cohen et al. (2014), who found better memory for displays for mixed-categories (faces and scenes) than for single categories. They attributed this effect to increased cortical space engaged by multiple categories. Because retinal size does not change which cortical regions process objects, our findings do not challenge cortical resource theory. Instead, they suggest that when size is task-irrelevant, heterogeneity does not enhance VWM for objects or colors.

A clue to the advantage for retinally larger objects comes from Experiment 3, where large color patches showed no memory advantage over small patches when display sizes were uniform. Because retinal size did not significantly affect color VWM, this argues against the idea that a larger change signal aided comparison. The advantage for objects in large photos therefore likely reflects effects on the encoding and retention. A key distinction is that objects are defined by visual detail, whereas colors are uniform. Larger photos may increase the information available to VWM, facilitating object encoding and preserving image resolution even as fidelity declines from perception to memory.

With regard to canonical size, small objects were remembered better than big objects, irrespective of retinal size. This advantage could reflect several factors, including presentation closer to real-life size on a screen, manipulability, or greater everyday familiarity. Additionally, category-linked memorability differences may also contribute (e.g., faces vs. scenes; Isola et al., 2011; Masarwa et al., 2022). Regardless, we found no evidence for a congruency effect between canonical and retinal size. Although congruency affects VLTM (Konkle & Oliva, 2011), canonically small objects were remembered better in the VWM task even when presented in large photo sizes. These findings suggest that VWM is more strongly tied to sensory and episodic experiences rather than to canonical size.

Our findings raise questions about whether VWM for shapes is always influenced by retinal size. Unlike objects, whose fidelity depends on visual detail, the surface properties of words are poorly retained once meaning is extracted. As long as words are large enough for reading, retinal size may have less impact on VWM for words than for objects. If an optimal retinal size for reading exists, very large words might even be remembered less well than smaller ones, meaning using only two retinal sizes may have limited generalizability of our findings. Moreover, in natural viewing, viewing distance likely interacts with retinal and canonical size, a factor not examined here. Future studies testing additional categories, size ranges, and paradigms incorporating viewing distance may clarify retinal-size effects.

Overall, our findings suggest that irrelevant sensory properties of a visual scene, such as retinal size, can influence VWM. Retinally larger objects show a memory advantage, likely due to greater engagement of sensory neurons during encoding, and they also outcompete smaller images in mixed-size displays. These results also have practical implications: billboards, advertisements, and websites may be more memorable when they use large images—even when depicting canonically small objects.

## Data Availability

These experiments were not preregistered. De-identified subject level data in an aggregated format, scripts, and Supplemental Materials containing additional analyses are available at the Open Science Framework (https://osf.io/hwstm/).
